# Anion Gap and Ionised Calcium as Diagnostic Indicators in Calves with Atresia Coli from Twenty-Two Cases

**DOI:** 10.3390/vetsci12111033

**Published:** 2025-10-24

**Authors:** Muhammed Kaan Yönez, Emre Tüfekçi, Umut Alpman, Gencay Ekinci

**Affiliations:** 1 Department of Surgery, Faculty of Veterinary Medicine, Erciyes University, Kayseri 38039, Türkiye; kaanyonez@erciyes.edu.tr; 2 Department of Wild Animal Diseases, Faculty of Veterinary Medicine, Erciyes University, Kayseri 38039, Türkiye; emretufekci@erciyes.edu.tr; 3 Department of Internal Medicine, Faculty of Veterinary Medicine, Erciyes University, Kayseri 38039, Türkiye; gekinci@erciyes.edu.tr

**Keywords:** anion gap, atresia, calcium, calves, coli

## Abstract

**Simple Summary:**

Intestinal atresia is a congenital disorder in calves where a segment of the intestine is closed or absent, causing digestive obstruction. Affected calves usually show abdominal swelling, lack of fecal output, and reduced appetite within the first days of life. In this study, we found that calves with atresia coli exhibited significantly elevated anion gap and plasma lactate, along with decreased ionised calcium and pH, compared to healthy calves. Anion gap and ionised calcium showed high diagnostic accuracy, suggesting their utility in distinguishing atresia coli from other neonatal conditions such as sepsis or diarrhea. Better understanding and timely management of intestinal atresia can improve animal welfare and reduce economic losses in cattle production.

**Abstract:**

This study aimed to evaluate blood lactate, anion gap, and ionised calcium levels as potential diagnostic biomarkers in calves with atresia coli, and to identify possible predisposing factors such as breed, gender, age, method of conception, number of lactations, and births. The study included twenty-two calves with atresia coli and ten healthy controls, all aged 1–11 days (median, 3 days), brought to Erciyes University Veterinary Faculty from Kayseri and nearby provinces due to non-defecation and abdominal swelling. Prominent clinical findings among the 22 calves with atresia coli included abdominal distension in 90.9%, anorexia in 81.8%, and depressed general posture in 86.4%. Blood gas analysis revealed significantly elevated lactate and anion gap and decreased ionised calcium and pH in atresia coli calves compared to controls (*p* < 0.05). Anion gap (>14.05 mmol/L) and ionised calcium (<1.205 mmol/L) demonstrated high diagnostic accuracy (AUC: 0.964 and 0.872, respectively), suggesting their potential as supportive biomarkers for early detection of atresia coli. The study data revealed that male gender, artificial insemination, and calves born from the third or subsequent pregnancies are statistically significant risk factors for the development of atresia coli. Atresia coli in calves is characterized by specific clinical signs and significant changes in blood gas parameters, such as elevated lactate and anion gap and reduced ionised calcium and pH. Early detection using these markers can improve diagnosis, and further studies should focus on prevention by addressing these risk factors.

## 1. Introduction

Atresia coli (AC) is a rare but serious congenital anomaly typically seen in newborn calves, characterised by congenital closure or underdevelopment of a part of the large intestine, leading to severe clinical signs and resulting in significant economic losses due to high treatment costs and calf mortality [1]. Although intestinal atresia in calves is generally considered a congenital disorder with low heritability (estimated at 0.0875), several environmental or management-related factors have been suggested. Among these, transrectal palpation before 42 days of gestation, particularly using the membrane slip technique, may act as a potential causal factor [2]. Additionally, male gender, artificial insemination, and calves born from the third or subsequent pregnancies were identified as significant risk factors in this study. Lesions typically occur in the spiral colon but may also affect the ascending, transverse, or descending colon [3]. Early diagnosis is critical for improving survival rates, reducing economic impact, and enabling timely surgical correction before the onset of irreversible complications.

In Türkiye, the incidence of atresia coli in calves has been reported to range between 1–10%, while rates of 4–9% have been observed among cases admitted to Veterinary Teaching Hospitals (VTH) [4,5,6,7,8,9]. Globally, prevalence in VTH-admitted calves ranges from 0.3–7% [10,11,12,13], because the mentioned interval comprises the following specific rates that you have mentioned. Atresia coli cases are predominantly seen in Holstein-Friesian calves, which have a higher risk than other breeds [14,15]. Although genetic factors are considered, the presence of sporadic cases, involvement of various breeds and regions, and occurrence in only one of identical twins suggest non-genetic influences [16]. A male predominance has been noted, with 76% of affected calves being male [1,17]. The condition is less frequent in calves from younger cows [15]. Polat [8] also noted a higher rate of congenital anomalies in calves conceived via artificial insemination compared to natural mating.

Atresia coli typically presents within the first week of life with signs such as absence of feces despite a patent anus, abdominal distension, anorexia, and depression [16,18]. Diagnosis is based on clinical findings and supported by imaging techniques like ultrasonography and radiography, though these may be inconclusive in early or nonspecific cases. In such instances, exploratory laparotomy remains the definitive diagnostic method [19,20,21]. Given the limitations of current diagnostic tools, there is a growing need for reliable biochemical markers to facilitate earlier and more accurate diagnosis in suspected cases.

The anion gap is a commonly used parameter in the assessment of metabolic acidosis and serves as an indirect indicator of tissue perfusion abnormalities and intestinal ischemia, both of which play a central role in the pathophysiology of atresia coli [21,22]. Elevated anion gap levels in affected calves often reflect anaerobic metabolism and lactic acidosis associated with severe abdominal distension and mesenteric circulation impairment [23]. On the other hand, ionised calcium plays a critical role in muscle contraction, membrane stability, and overall metabolic balance [15]. Hypocalcaemia is frequently reported in various perinatal diseases and may exacerbate systemic dysfunction by impairing neuromuscular and cardiovascular responses [23,24]. Despite the clinical importance of both parameters, studies specifically evaluating anion gap and ionised calcium levels in calves with atresia coli are notably limited. Investigating these biochemical markers may provide valuable insights into the metabolic disturbances associated with the disease and aid in early diagnosis and prognosis.

Research on novel biomarkers for diagnosing atresia coli is ongoing [25]. Markers such as intestinal and liver-type fatty acid-binding proteins (FABP) indicate intestinal injury and ischemia, while intestinal alkaline phosphatase supports mucosal defense [26]. Blood tests, including complete blood count and gas analysis, help assess systemic effects and prognosis [27], with common findings like leukocytosis, neutrophilia, and hypogammaglobulinaemia [28]. Elevated lactate, leukocytes and neutrophil counts have been reported in affected calves [21,25]. These parameters, alongside imaging and vital signs, aid rapid clinical and metabolic assessment.

This study aimed to evaluate hematology and blood gas parameters as potential diagnostic indicators in calves with atresia coli, and to identify possible predisposing factors such as breed, gender, age, method of conception, number of lactations, and births.

## 2. Materials and Methods

### 2.1. Study Design

The study was conducted as observational retrospective study. The STROBE checklist guidelines were adhered to in the preparation of this work.

### 2.2. Ethics Statement

The study was approved by the HADYEK, the Local Ethics Committee for Animal Experiments at Erciyes University (Approval No. 25/006). Animal owners consented to the participation of their animals in the study.

### 2.3. Selection and Description of Subjects

The material for this study consisted of 22 calves of different breeds and sexes diagnosed with atresia coli, and 10 healthy Holstein calves (5 males, 5 females) selected as the control group. All calves with atresia coli were 1 to 11 days old and brought to Erciyes University Veterinary Teaching Hospital from farms in Kayseri and neighboring provinces with complaints of “no defecation since birth” and “abdominal distension.” A series of questions was posed to all calf owners, seeking information regarding the breed, age, and sex of the calves, as well as the breed, number of births, and method of conception of the mothers. Healthy controls were obtained from the Veterinary Teaching Farm (VTF) and were randomly selected from clinically healthy calves aged 1–10 days. Calves included in the control group were raised under similar housing and feeding conditions from birth, were selected for comparable age ranges, and their mothers’ parity numbers were recorded. The study included calves from breeding cows and known semen sources used in artificial insemination. This approach minimized the potential effects of environmental and maternal differences, allowing for more reliable comparisons between atresia coli cases and healthy calves and enabling evaluation of the findings independent of external factors.

### 2.4. Physical Examination

A physical examination of the calves was performed in accordance with standard clinical procedures. General appearance, behaviour, posture and level of consciousness were evaluated during the examination. Vital signs, such as body temperature, heart rate, and respiratory rate, were measured using a digital thermometer and a stethoscope. Hydration status was assessed based on skin turgor, enophthalmos and moisture status of mucous membranes. Respiratory and gastrointestinal sounds were auscultated in the thoracic and abdominal regions. The presence of pain, distension or abnormal masses was investigated by palpation of the abdomen. Sucking reflex, defecation status, and any abnormal discharge (e.g., nasal, eye, or umbilical discharge) were recorded. In addition, signs of congenital anomalies or systemic diseases (e.g., depression, side-lying position, coma) were carefully observed and documented.

### 2.5. Blood Examination

After clinical examination, blood samples were collected from the jugular veins of calves for blood gas and hematological analyses to assess fluid-electrolyte balance and infection status. Samples for hematology were taken in K2-EDTA tubes (BD Vacutainer, Becton Dickinson, Franklin Lakes, NJ, USA) and for blood gas in lithium heparin syringes (Genject, Ankara, Türkiye) Analyses were performed within 5 min at Erciyes University Veterinary Teaching Hospital using an Exigo EosVet (Boule Medical AB, Stockholm, Sweden) hematology analyzer and an ABL80 FLEX blood gas analyzer (Radiometer Medical ApS, Bronshoj, Denmark). K2-EDTA samples were mixed at 40 rpm for 3 min before testing.

### 2.6. Laboratory Data

Data were obtained retrospectively from the hospital information system (Patient Registration System, ERUVetO; V.15042019/2015, Kayseri, Türkiye) between 1 December 2021 and 31 May 2023. Information such as the date the calves were brought to the hospital, management type, sex, clinical findings, blood gas and complete blood count (CBC) test results was obtained from this system.

Strong ion difference (SID) calculation was based on the sum of electrolyte concentrations in blood serum (sodium [Na^+^] + potassium [K^+^] − chloride [Cl^−^]) [29].

### 2.7. Diagnostic Imaging

**Figure 1:**
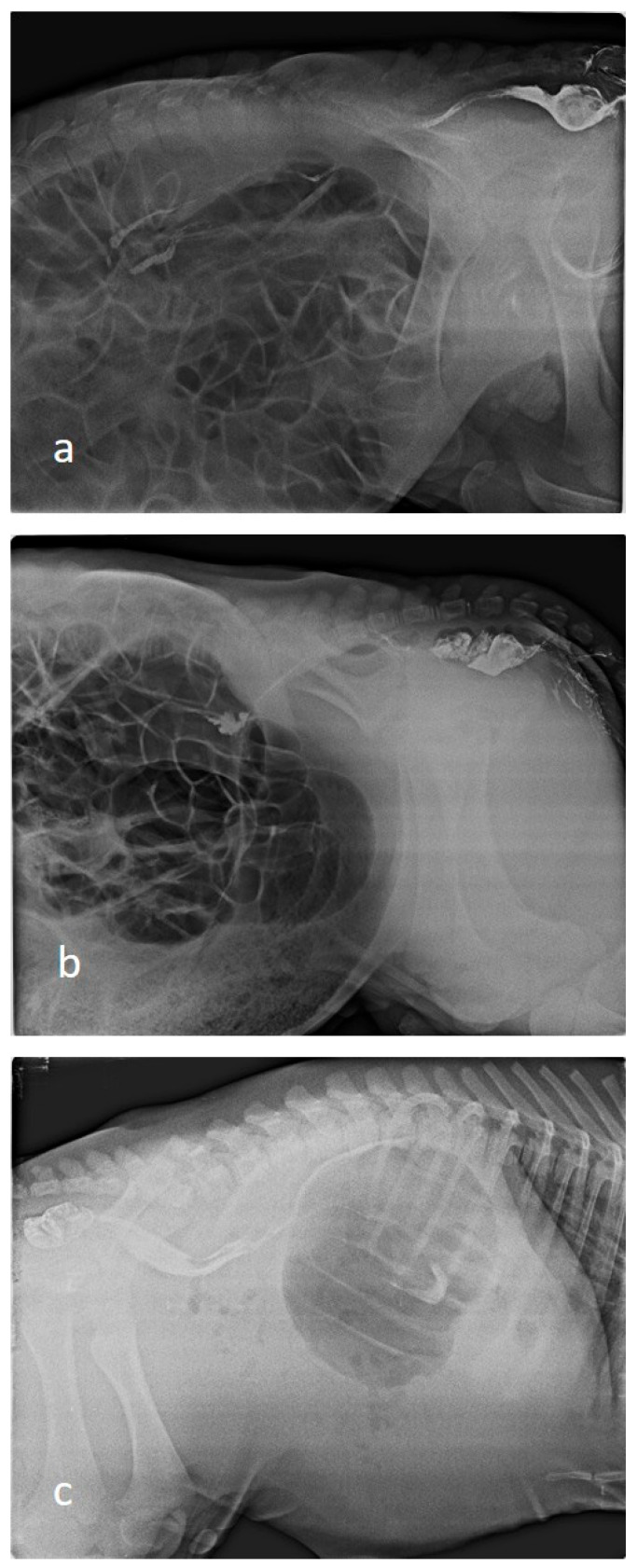
Indirect radiographic images of calves ((**a**); Type I, (**b**,**c**); Type III).

Indirect radiography was performed in all calves by rectal administration of barium sulphate contrast agent. Barium sulphate (E-Z-HD Barium sulphate, 98% powder, 340 g, OPAKIM, İstanbul, Türkiye) was diluted 1:3 with water and administered at a dose of 1 mL/kg via rectal probe [30]. The blind ends of the colon or rectum were radiographically visualised after barium administration (Figure 1). Barium-contrast radiography also showed the blind part of the colon. Indirect radiography was performed in the latero-lateral position using a 70 kilovolt (kV) and 150 milliampere (mA) setting on an X-ray machine (BLD-150AJ, AJEX Meditech Ltd., Seoul, Republic of Korea) with Fujifilm Computed Radiography (CR-IR 392, Fujifilm, Shanghai, China).

### 2.8. Postmortem Examination (Necropsy)

**Figure 2:**
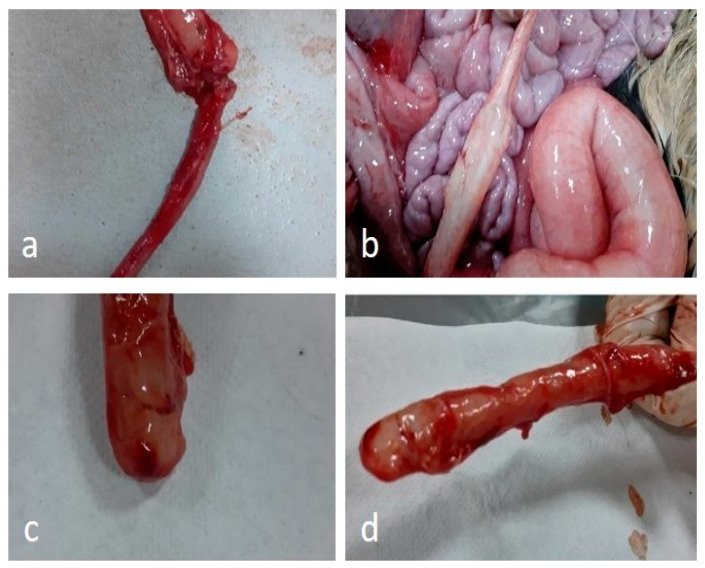
Calf necropsy images ((**a**,**b**); Type I, (**c**,**d**); Type III).

Necropsy was performed as soon as possible in calves euthanised with suspected atresia coli. The calf was placed in the supine (dorsal) position. In a disinfected area, the ventral midline incision was made carefully due to distension, and the skin and subcutaneous tissues were opened systematically. The thoracic and abdominal cavities were then carefully examined, and the organs were removed and macroscopically evaluated. The entire gastrointestinal tract was examined, extending from the mouth to the anus, while preserving anatomical integrity. Blind end or luminal obliteration was detected throughout the colon (Figure 2). The calves with atresia coli included in the study were classified into four types: type I, characterized by a thin membrane obstructing the lumen; type II, featuring blind ends connected by a fibrous cord; type III, complete separation with a mesenteric defect; and type IV, characterized by multiple atretic segments. In type IV, multiple atretic regions caused luminal obstruction and proximal dilation, with minimal changes in the surrounding mesentery or peritoneum [3].

### 2.9. Statistical Analysis

Statistical analyses were performed using SPSS 25.0 software (SPSS Inc., Chicago, IL, USA). The conformity of the data to a normal distribution was evaluated using the Shapiro–Wilk test and Q-Q plot. To determine whether the difference between the study and healthy control groups was statistically significant, an independent sample t-test or Mann–Whitney U test was applied, depending on the distribution characteristics of the data. Data were presented as mean ± standard deviation (mean ± SD) when the normality assumption was met and as median (minimum-maximum) values when the normality assumption was not met. The relationship between categorical variables was evaluated by the Pearson chi-square (χ^2^) test. Graphical representations were created with GraphPad Prism 9.3.1 software. In addition, ROC analysis was performed to determine the diagnostic accuracy and the optimum cut-off point was determined using the Youden index. The statistical significance level was set at *p* < 0.05.

## 3. Results

From 1 December 2021 to 31 May 2023, a total of 1283 calves from Kayseri and adjacent provinces (Sivas, Nevşehir, Yozgat, Niğde, Kırşehir) were admitted to the Erciyes University Faculty of Veterinary Teaching Hospital, with 22 cases (2.9%) diagnosed with clinical manifestations of atresia coli (Hospital Information System, ERUVetO; V.15042019/2015, Türkiye). As a result, 22 calves diagnosed with atresia coli were included in the study. The animal material of this study consisted of 22 newborn calves of different sexes (20 males, 2 females) and breeds (18 Holstein, 3 Simmental, 1 Brown) diagnosed with atresia coli. The median age of the calves was 4.2 days (min–max: 1–11 days). It was determined that 63.6% (14/22) of these calves were from traditional farms, and 36.4% (8/22) were from modern farms. All 22 calves diagnosed with atresia coli were euthanised. Among them, types III (63.6%), I (31.8%), and II (4.5%) atresia coli were diagnosed in 14, 7, and 1 calf, respectively.

### 3.1. Physical Examination Findings

Mean/median body temperatures, respiratory rate, and heart rate of the case group were 38.4 °C (interquartile range [IQR], 36.8–38.6; range, 32.0–39.7), 34/min (IQR, 24–44; range, 16–120) and 120 bpm (IQR, 100.00–128.00; range, 20–240), respectively.

Mean/median body temperatures, respiratory rate, and heart rate of the control group were 38.8 °C (interquartile range [IQR], 38.6–39.1), 49/min (IQR, 37–59) and 125 bpm (IQR, 112.50–140.25), respectively.

Statistically significant differences were found between case and control groups in terms of body temperature and respiratory rate (*p* < 0.05 and *p* < 0.01, respectively). Body temperature (38.26 ± 0.70 °C) and respiratory rate (34.18 ± 6.73 bpm) were notably lower in the case group compared to the control group (38.80 ± 0.33 °C and 49.00 ± 10.42 bpm). However, no significant difference was observed between the groups in terms of heart rate (*p* = 0.576).

Dehydration levels were normal in 22.7% (5/22), mild in 18.2% (4/22), moderate in 36.4% (8/22) and severe in 22.7% (5/22). The general condition was mild in 13.6% (3/22), moderate in 36.4% (8/22), severe in 40.9% (9/22), and comatose in 9.1% (2/22).

In the calves included in the study, anorexia 81.8% (18/22), mucous faeces 40.9% (9/22), bloody mucous faeces 27.3% (6/22), absence of faeces 31.8% (7/22), abdominal pain 36.4% (8/22) (severe = 6, mild = 2), abdominal distension 90.9% (20/22), weak sucking reflex 36.4% (8/22), absence of sucking reflex 72.7% (16/22), weakness 68.2% (15/22), enophthalmos/dehydration 77.3% (17/22), slow response to approach 36.4% (8/22), unresponsiveness to approach 50% (11/22), weak and exhausted appearance 86.4% (19/22), comatose state 9.1% (2/22), sternal recumbency position 45.5% (10/22) and side-lying position 9.1% (2/22) were recorded.

### 3.2. Complete Blood Count Findings

**Figure 3:**
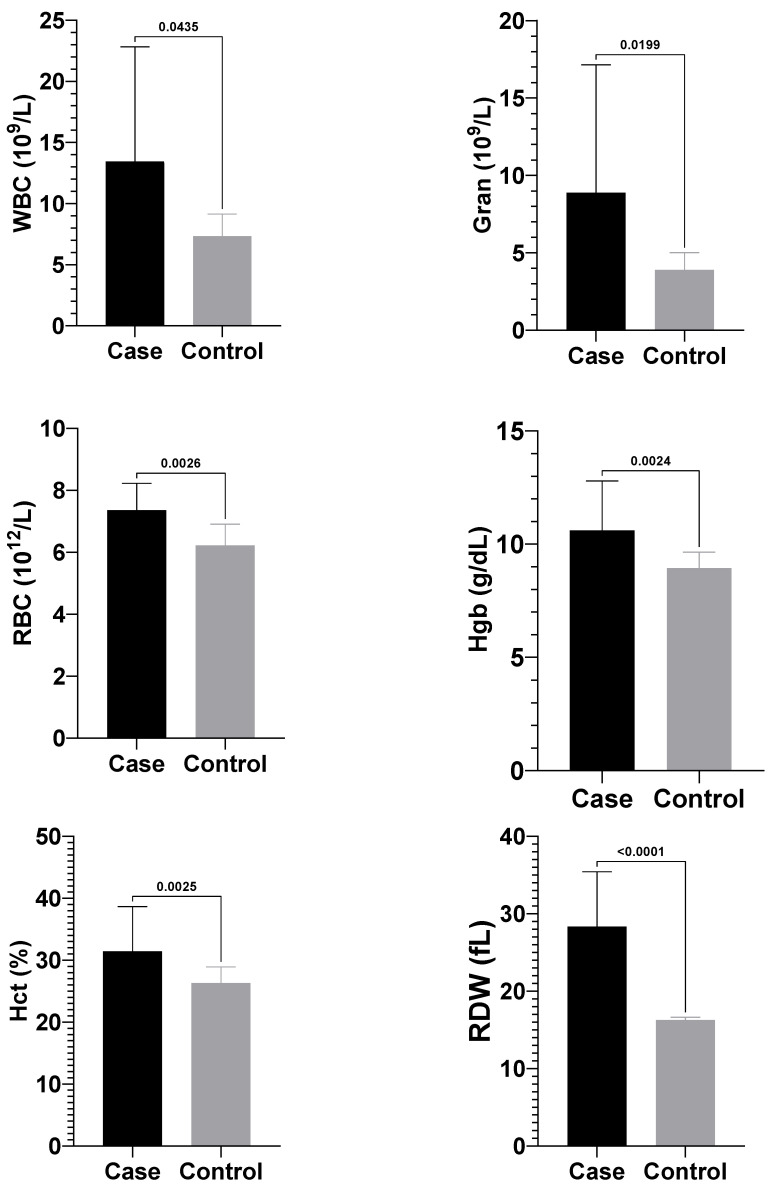
Comparison of complete blood count and vital parameters between the groups. WBC, white blood cell; Gran, granulocyte; RBC, red blood cell; Hgb, haemoglobin; Hct, haematocrit; RDW, red cell distribution width.

Calves with atresia coli showed significantly higher mean values of white blood cells (WBC) [11.0 (8.15–17.03) × 10^9^/L], granulocytes (Gran) [6.95 (3.75–11.13) × 10^9^/L], red blood cell (RBC) (7.39 ± 0.87 × 10^12^/L), haemoglobin (Hgb) (10.60 ± 2.19 g/dL), haematocrit (Hct) (31.41 ± 7.25%), and red cell distribution width (RDW) (28.34 ± 7.10 fL) compared to healthy calves, which had WBC [7.30 (5.85–8.12) × 10^9^/L], granulocytes [3.80 (3.25–4.92) × 10^9^/L], RBC (6.21 ± 0.69 × 10^12^/L), haemoglobin (8.93 ± 0.71 g/dL), haematocrit (26.30 ± 2.62%), and RDW (16.28 ± 0.35 fL) (*p* < 0.05) (Figure 3). No significant differences were observed between the groups for other complete blood count parameters.

**Figure 4:**
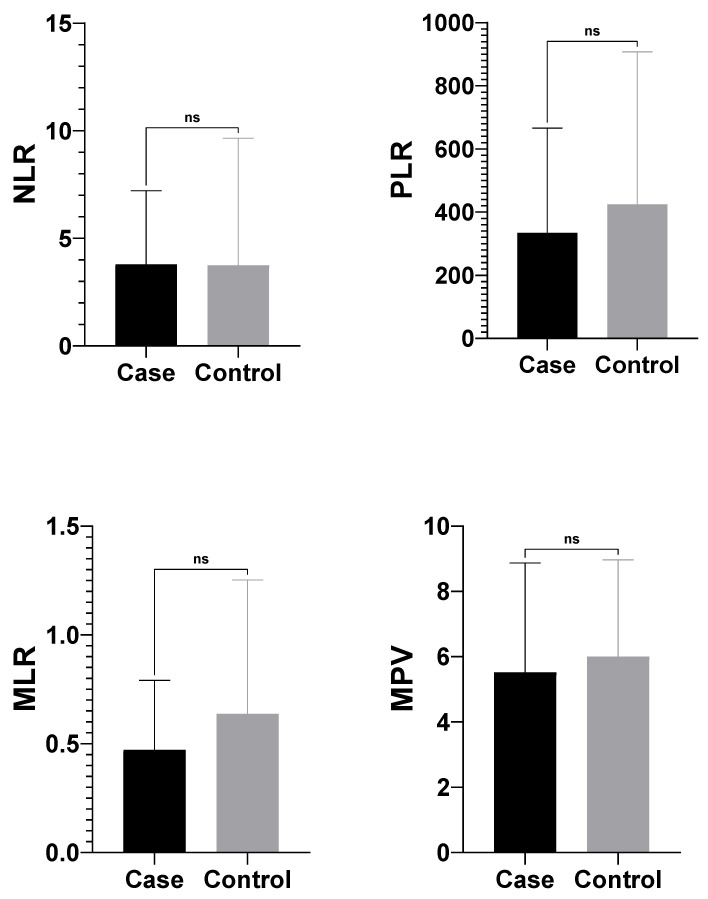
Comparison of complete blood count and vital parameters between the groups. WBC, white blood cell; Gran, Granulocyte; RBC, red blood cell; Hgb, haemoglobin; Hct, haematocrit; RDW, red cell distribution width. MLR: Monocyte-to-lymphocyte ratio, NLR: Neutrophil-to-Lymphocyte Ratio, MPV: Mean Platelet Volume, PLR: Platelet-to-Lymphocyte Ratio, ns: not significant.

In this study, the differences between neutrophil-to-lymphocyte tatio (NLR), platelet-to-lymphocyte ratio (PLR), monocyte-to-lymphocyte ratio (MLR) and mean platelet volume (MPV) values, which are some hematologic inflammation indicators, in neonatal calves diagnosed with atresia coli and healthy calves were investigated. According to the findings, there was no statistically significant difference between the case and control groups in all four parameters (*p* > 0.05). There was no difference between calves with atresia coli and healthy calves in terms of haematological index calculations (Figure 4).

### 3.3. Blood Gas Analysis Findings

**Figure 5:**
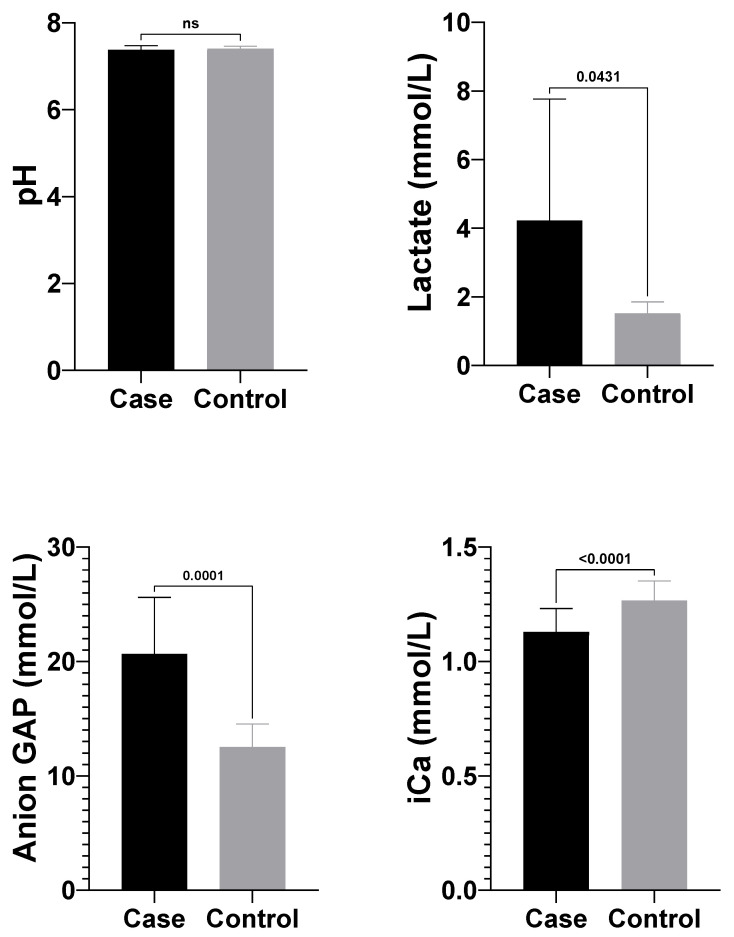
Comparison of pH, Lactate, Anion Gap and iCa^2+^ parameters between case and control groups. pH; Power of Hydrogen, iCa^2+^; Ionised calcium, ns: not significant.

Significant differences in blood gas parameters between atresia coli and healthy calves are shown in Table 1. Lactate (4.05 ± 3.54 mmol/L) and anion gap (25.35 ± 7.64 mmol/L) levels in the atresia coli group calves were significantly higher than lactate (1.53 ± 0.33 mmol/L) and anion gap (12.53 ± 2.02 mmol/L) levels in healthy calves. On the other hand, pH (7.38 ± 0.10) and ionised calcium (Ca^2+^) (1.13 ± 0.10 mmol/L) levels in the atresia coli group were significantly lower compared to the healthy group (pH: 7.43 ± 0.03; Ca^2+^: 1.30 ± 0.04 mmol/L) (Table 1) (Figure 5).

**Figure 6:**
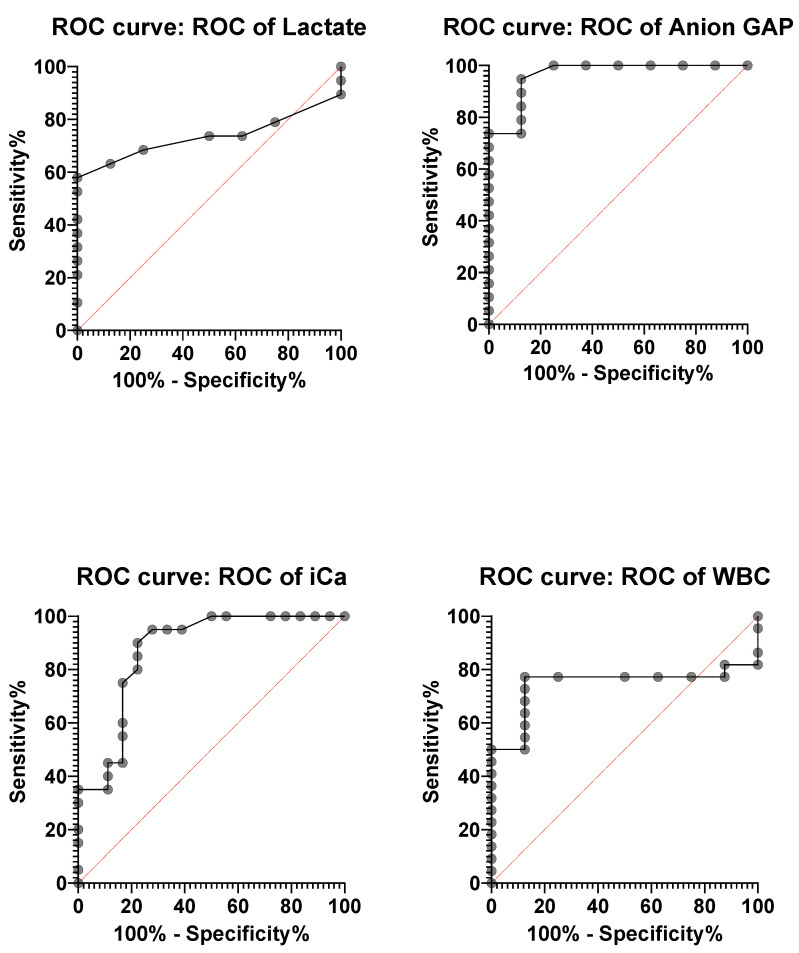
ROC analyses of lactate, Anion Gap, iCa^2+^, and WBC levels in the diagnosis of atresia coli. AUC values were found to be 1.00 for lactate, 0.979 for Anion Gap, 0.611 for iCa^2+^, and 0.531 for WBC, respectively.

In this study, the diagnostic accuracy of lactate, anion gap, ionised calcium (iCa^2+^), and white blood cell (WBC) counts was evaluated using receiver operating characteristic (ROC) curve analysis (Figure 6). Among the markers analyzed, anion gap demonstrated the highest diagnostic performance, with an area under the ROC curve (AUC) of 0.964 (95% CI: 0.893–1.000; *p* < 0.001). The optimal cut-off value of >14.05 mmol/L yielded a sensitivity of 94.74% (95% CI: 75.36–99.73) and a specificity of 87.50% (95% CI: 52.91–99.36), with the highest Youden’s index of 0.82, indicating strong diagnostic utility (Table 2).

Ionised calcium (iCa^2+^) also showed excellent discriminative ability, with an AUC of 0.872 (95% CI: 0.754–0.990; *p* < 0.001). The cut-off point of >1.205 mmol/L provided a sensitivity of 75.00% and a specificity of 83.33%, supporting its potential as a valuable biomarker (Table 2).

WBC count displayed a moderate diagnostic value with an AUC of 0.744 (95% CI: 0.566–0.923; *p* = 0.044). A cut-off value of >8.650 × 10^9^/L yielded a sensitivity of 77.27% and specificity of 87.50%, with a Youden’s index of 0.65, suggesting its supplementary role in diagnosis (Table 2).

Lactate, with an AUC of 0.733 (95% CI: 0.548–0.919), approached but did not reach statistical significance (*p* = 0.059). Although it showed good specificity (87.50%), its lower sensitivity (63.16%) and a relatively modest Youden’s index (0.51) indicate limited standalone diagnostic utility (Table 2).

In the comparison of calves with different types of atresia coli (Type 1 vs. Type 3), no statistically significant differences were observed in NLR (2.33 ± 1.30 vs. 4.76 ± 3.89; *p* = 0.322), PLR [215.42 (49.52–295.00) vs. 331.43 (203.08–390.69); *p* = 0.197], or MPV (5.80 ± 3.53 vs. 5.50 ± 3.48; *p* = 0.856) values. However, MLR was significantly higher in calves with Type 3 atresia coli (0.58 ± 0.35; 0.55 [0.30–0.84]) compared to those with Type 1 (0.29 ± 0.10; 0.28 [0.20–0.38]) (*p* = 0.031) (Table 3).

### 3.4. Radiographic Findings

In cases of atresia coli, radiographic evaluation revealed that the intestinal passage terminated in a distal segment, resulting in severe proximal distension. In abdominal radiographs of calves, it was observed that the spiral colon and cecum were markedly distended with gas and liquid content, whereas distal colonic segments were free of gas and content. An enlarged gas-filled intestinal anus, especially in the left flank region, was typical. Contrast-enhanced radiography revealed that the contrast had travelled up to a certain level, and no passage distal to the atretic segment was observed.

### 3.5. Postmortem Findings

Proximal intestinal distension, including the cecum, spiral colon, and sometimes the ileum segments, were notably filled with gas, fluid, and meconium. Wall thinning and overstretching were common in these segments. The atretic segment was typically situated in the distal part of the spiral colon. Collapse and absence of contents were observed in the distal colon. In some calves, secondary changes such as atelectasis in the lungs, passive congestion in the liver, and right-sided dilatation of the heart occurred due to abdominal distension pushing the diaphragm cranially.

### 3.6. Predisposing Factor Findings

Pearson’s chi-square analysis revealed a statistically significant association between male gender and the presence of atresia coli (χ^2^ = 7.58, *p* = 0.006). In addition, a statistically significant association was found between calves born via artificial insemination and the occurrence of atresia coli (χ^2^ = 5.86, *p* = 0.015). It should be noted that in artificially inseminated cows, pregnancy diagnosis via transrectal palpation with the membrane slip technique is often performed more frequently than in naturally mated cows, which could represent a potential confounding factor. Finally, a statistically significant relationship was determined between calves born after three or more pregnancies and the presence of atresia coli (χ^2^ = 6.48, *p* = 0.011).

## 4. Discussion

The prevalence of atresia coli in calves varies considerably depending on the geographical region, genetic factors and environmental conditions [12]. A prevalence of 0.76% was reported in the United States of America [10], 6.9% in Israel [11], and 0.35% in Germany [13]. In Türkiye, the prevalence of atresia coli in calves brought to VTH between 2003 and 2024 was reported to be between 0.90% and 9.09% [4,5,6,7,8,9]. In the present study, atresia coli was diagnosed in 22 (1.71%) of 1.283 calves brought to VTH between 2021 and 2022. Although the rate obtained in the present study is higher than the rate reported by Karasu et al. [9], it is lower than some studies in different regions of Türkiye; it is also higher than the rates reported in the USA [10] and Germany [13] and lower than in Israel [11].

The anion gap is an important parameter for categorising causal factors in acid-base imbalances and determining prognosis [12,22]. In the current study, it was observed that the anion gap (mmol/L) values measured in calves with atresia coli were compatible with the findings reported by Yildiz et al. [21] and were significantly higher when compared with reference intervals [31]. This coincides with Trefz et al. [23] description of metabolic acidosis with high anion gap in calves due to diarrhea and other intestinal disorders. Similarly, Dillane et al. [29] also drew attention to this pathophysiological process. The elevated anion gap level in calves with atresia coli is mainly associated with lactic acidosis caused by tissue hypoxia due to intestinal obstruction. Increased lactate and other acidic anions cause the anionic gap to increase in metabolic acidosis. This is also favoured by bicarbonate consumption.

Hypocalcaemia observed in calves with atresia becomes more pronounced, especially in cases accompanied by decreased ionised calcium (iCa^2+^) levels, hypothermia and severe dehydration [32]. This may have negative effects on muscle contractility, cardiac function and neuromuscular transmission [24]. Ionised calcium levels depend not only on total calcium but also on the balance with other electrolytes such as serum pH, albumin level and magnesium. Indeed, fluid losses, electrolyte imbalances and circulatory disorders due to atresia coli may cause an imbalance in iCa^2+^ levels [15]. In the present study, pH (7.38 ± 0.10) and Ca^2+^ (1.13 ± 0.10 mmol/L) values were found to be significantly lower in atresia calves compared to the healthy control group (pH: 7.43 ± 0.03; BE: 1.30 ± 0.04 mmol/L), which supports this literature information. In addition, pathophysiological processes commonly observed in patients with atresia, such as metabolic acidosis, endotoxaemia and systemic inflammatory response, have been reported to contribute to the decrease in iCa^2+^ levels [23,24]. In line with these findings, the decrease in ionised calcium levels may be considered a supportive parameter in terms of diagnosis and prognosis in atresia calves.

The anaerobic metabolic pathway known as glycolysis is the first step in glucose metabolism and occurs in the cytoplasm of almost all cells. The end product of this pathway, pyruvate, is metabolised to lactate by the enzyme lactate dehydrogenase [33]. Normal blood lactate level in healthy calves is considered to be 0.5–2.0 mmol/L [34]. In the present study, blood lactate level was found to be significantly higher in calves with atresia coli compared to healthy calves, in accordance with the findings of Yildiz et al. [21] and Coşkun et al. [35]. The possible mechanism that may explain our findings is the increase in blood lactate levels in calves with atresia coli, resulting from tissue hypoxia caused by intestinal obstruction and accompanying anaerobic metabolism. This increase is associated with pathological processes such as intestinal distension, circulatory disturbance and possible necrosis and is also supported by metabolic acidosis.

Both septicemia [36] and rumen drinking [37,38] may lead to a marked increase in plasma lactate concentration and anion gap due to tissue hypoxia and metabolic acidosis, and therefore should be considered as possible differential diagnoses. A similar conclusion was drawn by Stocker et al. [37] who found a high anion gap metabolic acidosis in 50 calves with chronic indigestion caused by ruminal drinking. In addition, serum ionised calcium concentration has been reported to be an important prognostic indicator in critically ill calves [39]. A serum ionised calcium level below 2.34 mEq/L (≈2.34 mmol/L) was associated with a reduced likelihood of survival, indicating that hypocalcaemia represents a poor prognostic factor [39].

Although abdominal distension and discomfort are often associated with tachycardia, the lack of a significant increase in heart rate observed in our study may be explained by several factors. Compensatory physiological mechanisms in neonatal calves, such as baroreflex-mediated autonomic adjustments and limited sympathetic activation, can maintain heart rate within normal ranges during early stages of distress [35]. Additionally, the severity and duration of clinical signs at the time of examination may have been insufficient to elicit marked tachycardia, as heart rate increases in calves generally correlate with the degree of systemic compromise rather than the mere presence of abdominal pathology [40]. Furthermore, hypoglycemia is known to influence cardiovascular responses and can blunt sympathetic-mediated tachycardia; however, blood glucose levels were not measured in our study, precluding assessment of its contribution. Future studies including glycemic evaluation may provide additional insights into how metabolic derangements, such as hypoglycemia, acidosis, and lactate accumulation, interact with cardiovascular responses in calves with atresia coli.

The changes observed in complete blood count parameters in calves with atresia coli are considered haematological reflections of obstruction, inflammation, dehydration and systemic stress. In the present study, the mean WBC [11.0 (8.15–17.03) × 10^9^/L] and granulocyte [6.95 (3.75–11.13) × 10^9^/L] counts in calves with atresia coli compared to healthy calves [WBC; 7.30 (5.85–8.12) × 10^9^/L; Gran: 3.80 (3.25–4.92) × 10^9^/L] were found statistically significantly higher than in healthy calves (*p* < 0.05). Elevated WBC and neutrophilia are commonly observed in cases with intestinal atresia, and plasma protein levels generally remain normal, although haemoconcentration is detected [25]. This finding aligns with previous studies. In the literature, it has been reported that WBC and neutrophil levels are higher in calves with atresia than in healthy calves [21,25,30]. Salci et al. [20] found leucocytosis in 40% and neutrophilia in 72% of the 22 cases they examined. The increase in WBC and granulocyte levels can be largely explained by mechanisms such as acute inflammatory response, the effect of stress hormones and bacterial translocation. In addition, RBC (7.39 ± 0.87 × 10^12^/L), Hgb (10.60 ± 2.19 g/dL), Hct (31.41 ± 7.25%) and RDW (28.34 ± 7.10 fL) values were significantly higher than in healthy calves in the current study (RBC; 6.21 ± 0.69 × 10^12^/L, Hgb; 8.93 ± 0.71 g/dL, Hct; 26.30 ± 2.62%, RDW; 16.28 ± 0.35 fL), suggest the effect of haemoconcentration, increased erythropoiesis against hypoxia and impaired circulatory balance on haematological parameters in atresia cases. In this context, complete blood count findings are important biomarkers to evaluate the systemic effects of the disease in calves with atresia coli.

In the present study, the mean RBC, haemoglobin (Hgb), and haematocrit (Hct) values of calves with atresia coli were significantly higher than those of healthy calves (*p* < 0.05). Haematological and biochemical changes, such as haemoconcentration (Hgb: 14.6 g/dL), dehydration (Hct: 51.3%), hypoproteinaemia (TP: 4.4 g/dL), and hypernatraemia (Na: 142 mEq/L), were reported in a calf with atresia coli [41]. While haematology and serum biochemistry values are generally normal during the first 48 h of the disease, dehydration develops as a result of fluid accumulation in the intestines and bacterial growth, which leads to an increase in haematocrit and total protein values. Over time, electrolyte irregularities such as azotemia, hypochloremia and hypokalemia become evident [42]. The increase in haemoglobin and haematocrit in calves with atresia coli is typically due to haemoconcentration resulting from dehydration caused by intestinal obstruction; the decrease in plasma volume increases erythrocyte density, leading to an increase in these parameters.

In one study, the mean platelet volume (MPV), platelet distribution width (PDWc), neutrophil (NEU), and neutrophil percentage (NEU%) values were significantly higher in the study group compared to the control group [25]. Obstruction of the intestinal passage in calves with atresia may cause impaired tissue perfusion and consequent intestinal necrosis or ischaemic changes. This leads to an increase in monocytes (monocytosis) and in MLR (Monocyte/Lymphocyte Ratio). Monocytes are activated in response to tissue damage and secrete proinflammatory cytokines. Intestinal distension and bacterial translocation in calves with atresia can lead to systemic infection and endotoxaemia. Monocytes are among the first cells to respond to endotoxins, which contributes to increased MLR [23].

Atresia coli is morphologically classified as type I: membranous atresia, type II: segments separated by a fibrous cord, and type III: segment separation with blunt ends. Type III is the most common form [32]. In the present study, unlike the findings of Salci et al. [20], the most common form in calves with atresia coli was type III (n = 14), followed by type I (n = 7) and type II (n = 1). Salci et al. [20] reported that the most common form was type IV (n = 9), followed by type II (n = 8) and type III (n = 5); moreover, type I atresia was not encountered.

Although there are studies reporting a higher incidence of atresia coli in male calves [1,17,43,44], there are also studies indicating that there is no significant relationship between gender and the occurrence of this anomaly [19]. In the present study, as a result of the Pearson chi-square analysis, it was determined that there was a significant relationship between male calves and atresia coli and male calves were found to be statistically significantly more likely to develop atresia coli. Although the exact reason is unknown, it is thought that differences in genetic and embryonic development processes may be a contributing factor. This finding suggests that sex may be an important factor in the susceptibility of calves to atresia coli disease.

Although artificial insemination allows for the selection of genetic traits, it may lead to the accumulation of certain genetic risk factors. In the current study, it was found that calves born through artificial insemination were significantly more likely to develop atresia coli (χ^2^ = 5.86, *p* = 0.015). However, it should be noted that in cows undergoing artificial insemination, pregnancy diagnosis is often performed more frequently using transrectal palpation with the membrane slip technique, which may act as a potential confounding factor [2]. Similarly, Polat [8] and Karasu et al. [9] reported that the frequency of congenital anomalies, especially atresia coli, increased in calves born through artificial insemination. These findings suggest that both genetic selection and potential management-related procedures, such as transrectal palpation, may play a role in the development of congenital anomalies, and it is recommended that high-risk genetic lines be identified and considered in breeding programs.

In the current study, the probability of atresia coli was found to be significantly higher in calves born after three or more pregnancies (χ^2^ = 6.48, *p* = 0.011). Studies such as those by Jubb [43] and Keane et al. [15] have also reported that intestinal atresia is less common in the offspring of young cows. The increased frequency of congenital anomalies with increasing number of pregnancies may be explained by factors such as reproductive system fatigue, immune weakness or age-related deterioration in the integrity of genetic material in the mother cow.

Congenital anomalies are structural or functional disorders that develop in the intrauterine period and present at birth [45]. Colonic atresia in calves is frequently associated with other anomalies such as atresia ani, tail deficiency, renal agenesis, umbilical lesions, cryptorchidism and spinal dysraphism [1]. In the current study, omphalitis was detected in one calf, and an umbilical hernia was present in the other. The presence of other congenital anomalies or infections in cases of atresia coli suggests that this disease is not typically seen in isolation and often co-occurs with additional health issues.

This study has certain limitations that should be acknowledged. First, as a retrospective and observational study, data were obtained from hospital records, which may have led to incomplete or inconsistent documentation of some clinical variables. Second, the sample size was relatively limited, particularly in the control group, which may restrict the generalizability of the findings to a broader calf population. Third, only calves presented to a single veterinary teaching hospital were included; therefore, regional or management-related differences that might influence the occurrence and characteristics of atresia coli could not be fully assessed. In addition, laboratory parameters were obtained at a single time point upon admission, preventing longitudinal evaluation of changes during disease progression or treatment. Finally, necropsy findings were limited to cases with a confirmed diagnosis of atresia coli, and histopathological confirmation was not performed in all cases. Despite these limitations, the study provides valuable clinical and laboratory insights into the characterization of atresia coli in neonatal calves under field and hospital conditions.

## 5. Conclusions

In conclusion, anion gap was found to be significantly higher and ionised calcium levels significantly lower in calves diagnosed with atresia coli compared to healthy calves, and it was evaluated that these parameters could be used as complementary tests in diagnosis. In addition, the data obtained in the study revealed that sex, artificial insemination and the number of pregnancies at birth may play an important role in the development of atresia coli. Accordingly, statistically significant risk factors for atresia coli were identified, including male sex, artificial insemination, and calves born from the third or subsequent pregnancies.

## Figures and Tables

**Table 1:** Comparison of complete blood count and vital parameters between groups.

Parameters	Atresia Coli (n = 22)	Healthy Calves (n = 10)	*p* Values
pH	7.38 ± 0.10	7.43 ± 0.03	0.595
pCO_2_ (mmHg)	43.64 ± 8.39	41.69 ± 3.19	0.381
pO_2_ (mmHg)	30.15 ± 3.77	28.75 ± 4.13	0.395
Na^+^ (mmol/L)	138.90 ± 8.55	139.13 ± 2.59	0.943
K^+^ (mmol/L)	4.40 ± 1.19	4.84 ± 0.26	0.132
iCa^2+^ (mmol/L)	1.13 ± 0.10	1.27 ± 0.09	<0.001
CI^−^ (mmol/L)	97.30 ± 3.99	95.38 ± 2.45	0.136
cLactate (mmol/L)	4.23 ± 3.54	1.53 ± 0.33	0.043
cHCO3^−^(P) (mmol/L)	26.89 ± 5.42	31.25 ± 4.18	0.052
BE (mmol/L)	2.99 (−8.3–11.6)	6.55 (−1.3–8.5)	0.842
Anion gap (mmol/L)	20.66 ± 4.94	12.53 ± 2.02	<0.001
SID	41.82 ± 16.31	48.59 ± 2.41	0.257

pCO_2_, partial pressure of carbon dioxide; pO_2_, partial pressure of oxygen; Na^+^, natrium; K^+^, Potassium; iCa^2+^, ionised calcium; CI^−^, chlorine; cLactate, concentration of L-lactate in plasma; cHCO3^−^(P), concentration of hydrogen carbonate in plasma (also termed actual bicarbonate); cBE, base excess in blood; SID, strong ion difference; NR, reference interval not reported.

**Table 2:** Sensitivity, specificity, likelihood ratio and area under the receiver operating Characteristics (ROC) curve values for Lactate, Anion Gap, iCa^2+^ and WBC cut-off points.

Marker	AUC	95% Cl		SE	*p* Values
Lactate (mmol/L)	0.733	0.548 to 0.919		0.095	0.059
Anion Gap (mmol/L)	0.964	0.893 to 1.000		0.036	<0.001
iCa^2+^ (mmol/L)	0.872	0.754 to 0.990		0.060	<0.001
WBC (×10^9^/L)	0.744	0.566 to 0.923		0.091	0.044
		**Sensitivity analysis**			
**Marker**	**Cut-off value**	**Sensitivity** **% (95% CI)**	**Specificity** **% (95% CI)**	**Max. Youden’s** **index**	
Lactate (mmol/L)	>1.950	63.16 (41.04–80.85)	87.50 (52.91–99.36)	0.51	
Anion Gap (mmol/L)	>14.05	94.74 (75.36–99.73)	87.50 (52.91–99.36)	0.82	
iCa^2+^ (mmol/L)	>1.205	75.00 (53.13–88.81)	83.33 (60.78–94.16)	0.58	
WBC (×10^9^/L)	>8.650	77.27 (56.56–89.88)	87.50 (52.91–99.36)	0.65	

AUC: Area under the ROC curve, Cl: Confidence Interval, SE; Standard error, AUC: Area under the ROC curve, Cl: Confidence interval, LR: Likelihood ratio, iCa^2+^; Ionised calcium, WBC; White blood cells.

**Table 3:** Comparison of Atresia coli parameters.

	Type 1 (n = 7)	Type 3 (n = 14)	*p* Value
NLR	2.33 ± 1.30	4.76 ± 3.89	0.322
PLR	215.42 (49.52–295.00)	331.43 (203.08–390.69)	0.197
MLR	0.29 ± 0.10 ^a^ 0.28 (0.20–0.38)	0.58 ± 0.35 ^b^ 0.55 (0.30–0.84)	0.031
MPV	5.80 ± 3.53	5.50 ± 3.48	0.856

NLR: Neutrophil-to-Lymphocyte Ratio, PLR: Platelet-to-Lymphocyte Ratio, MLR: Monocyte-to-Lymphocyte Ratio, MPV: Mean Platelet Volume, ^a,b^: Significant differences within type.

## Data Availability

The original contributions presented in this study are included in the article material. Further inquiries can be directed to the corresponding author.

## Abbreviations

The following abbreviations are used in this manuscript:

AC	Atresia coli
FABD	Fatty Acid-Binding Proteins
VTH	Veterinary Teaching Hospital
CBC	Complete Blood Count
SID	Strong Ion Difference
kV	Kilovolt
mA	Milliampere
